# Supplementary material for: Age-dependent virulence of human pathogens
Source: PLoS Pathog. 2022 Sep 22;18(9):e1010866. doi: 10.1371/journal.ppat.1010866 (PMC9531802; doi:10.1371/journal.ppat.1010866)
Supplement: S1 Table — For each disease, we report the pathogen, the pathogen type, the pathophysiology, the incubation period, the duration of symptoms/illness, the transmission route, the length of the human pathogen association, the clinical symptoms, whether the pathogen is known to have an animal reservoir, whether the pathogen can be transmitted from human to human. This information was used to attribute the values for each of the variables included in the finite mixture models. Sources from which this information was extracted are given at the bottom of the table. (DOCX) [file ppat.1010866.s001.docx]

S1 Table. List of human infectious diseases used to investigate age-specific case fatality rate (CFR). For each disease, we report the pathogen, the pathogen type, the pathophysiology, the incubation period, the duration of symptoms/illness, the transmission route, the length of the human pathogen association, the clinical symptoms, whether the pathogen is known to have an animal reservoir, whether the pathogen can be transmitted from human to human. This information was used to attribute the values for each of the variables included in the finite mixture models. Sources from which this information was extracted are given at the bottom of the table.

| **Disease** | **Pathogen** | **Pathogen type** | **Pathophysiology** | **Incubation period** | **Duration of symptoms/illness** | **Transmission route** | **Length of human-pathogen association** | **Clinical symptoms** | **Animal reservoir** | **Human-to-human transmission** |
| --- | --- | --- | --- | --- | --- | --- | --- | --- | --- | --- |
| Campylobacteriosis [1,2] | *Campylobacter* (currently 17 species, the most frequently reported are *C. jejuni* and *C. coli*) | Gram^-^bacteria | *C. jejuni* appears to invade and destroy epithelial cells. Some strains of *C. jejuni* produce a cholera-like enterotoxin, which is important in the watery diarrhoea observed in infections.  The organism produces diffuse, bloody, edematous, and exudative enteritis | The onset of disease symptoms usually occurs 2 to 5 days after infection with the bacteria, but can range from 1 to 10 days | The symptoms typically last 3 to 6 days | The main route of transmission is generally foodborne, via undercooked meat and meat products, as well as raw or contaminated milk. Contaminated water or ice is also a source of infection | The disease was first recognized by  Theodor Escherich in 1886 | The most common clinical symptoms of Campylobacter infections include diarrhoea (frequently bloody), abdominal pain, fever, headache, nausea, and/or vomiting | Campylobacter species are widely distributed in most warm-blooded animals | Campylobacteriosis is a zoonosis, a disease transmitted to humans from animals or animal products |
| Dengue [3-5] | Dengue is caused by a virus of the Flaviviridae family and there are four distinct, but closely related, serotypes of the virus that cause dengue (DENV-1, DENV-2, DENV-3 and DENV-4) | RNA virus | The illness begins abruptly and is followed by the three phases — the febrile phase, the critical phase, and the recovery phase. Instead of improving with the subsidence of fever, patients with increased capillary permeability may manifest with symptoms indicative of plasma leakage and enter what is termed the critical phase | The incubation period is 4–10 days after the bite from an infected mosquito | Symptoms usually last for 2–7 days | The virus is transmitted to humans through the bites of infected mosquitoes | The first record of a clinically compatible disease has been recorded in a Chinese medical encyclopaedia in 992 | Dengue virus infection has a wide spectrum of clinical manifestations, ranging from asymptomatic infection, to symptoms of non-severe disease (such as flu-like symptoms, fever, retro-orbital headach, fatigue, arthalgia, myalgia, nausea, vomiting, or lymphoadenopathy), and to severe complications including signs of plasma leakage (such as pleural effusion or ascites), hemorrhagic tendencies (such as petechiae, ecchymoses, purpura, easy bruising at venipuncture sites, mucosal bleeding, gastrointestinal bleeding, hematemesis, or melena), and organ failure associated with shock | Dengue has a sylvatic cycle from primates to humans | Human-to-human transmission occurs through mosquito bite. Maternal transmission is also possible |
| Hepatitis A [6,7] | Hepatitis A Virus (HAV) | RNA virus | Hepatitis A is a liver disease caused by the HAV | The incubation period of hepatitis A is approximately 28 days (range 15 to 50 days) | Clinical illness usually does not last longer than 2 months (although 10% to 15% of persons have prolonged or relapsing signs and symptoms for up to 6 months) | The hepatitis A virus is transmitted primarily by the faecal-oral route, when an uninfected person ingests food or water that has been contaminated with the faeces of an infected person | The first descriptions of hepatitis (epidemic jaundice) are generally attributed to Hippocrates | Symptoms of hepatitis A range from mild to severe and can include fever, malaise, loss of appetite, diarrhoea, nausea, abdominal discomfort, dark-coloured urine and jaundice | Humans are the only natural reservoir of the virus | HAV infection is acquired primarily by the fecal-oral route by either ingestion of contaminated food or water or direct contact with an infectious person |
| Pertussis [8] | *Bordetella pertussis* | Gram^-^bacterium | The bacterium produces toxins (virulence factors) that paralyze the cilia, and cause inflammation of the respiratory tract, which interferes with the clearing of pulmonary secretions.  Bordetella pertussis has tropism for pulmonary tissue and rarely disseminates to other organs | The incubation period is commonly 7-10 days with a range 4-21 days | Duration of the catarrhal stage is 1-2 weeks; duration of the  paroxysmal stage is 1-6 weeks (up to 10 weeks); duration of the  convalescent stage is 2-3 weeks | Transmission most commonly occurs person-to-person through contact with respiratory droplets, or by contact with airborne droplets of respiratory secretions | Outbreaks of pertussis were first described in the 16th century by Guillaume de Baillou | Catarrhal stage: coryza, sneezing, low-grade fever, mild occasional cough;  Paroxysmal stage: bursts of numerous rapid coughs (the patient may become cyanotic) | No animal or insect source or vector is known | Person-to-person transmission through contact with respiratory droplets |
| Salmonellosis [9,10] | *Salmonella enterica*, *S. bongori.*  The most common serotypes of Salmonella that cause human infection are Enteritidis, Typhimurium, Newport, and Javiana | Gram^-^ bacterium | Non-typhoidal Salmonella (NTS) usually cause self-limiting diarrhea | Symptoms usually begin 6 hours to 6 days after infection | Symptoms last 4-7 days | Salmonella is usually transmitted to humans by eating foods contaminated with small amounts of animal feces | Salmonella were first discovered by Daniel E. Salmon in 1885 | diarrhea, fever, stomach cramps | Salmonella live in the intestinal tracts of humans and other animals, including poultry and other birds, amphibians, and reptiles | An estimated 94% of salmonellosis is transmitted by food. Humans usually become infected by eating foods contaminated with feces from an infected animal. As a result, implicated foods are often of animal origin such as beef, poultry, milk, and eggs |
| Influenza [11-13] | Influenza virus.  There are four types of influenza viruses: A, B, C and D. Human influenza A and B viruses cause seasonal epidemics of disease. Influenza A viruses are the only influenza viruses known to cause flu pandemics | RNA virus | Influenza viruses infect the nose, throat, and sometimes the lungs | The time from when a person is exposed and infected with flu to when symptoms begin is about 2 days, but can range from about 1 to 4 days | Uncomplicated influenza signs and symptoms typically resolve after 3-7 days for the majority of people | Flu viruses spread mainly by tiny droplets made when people with flu cough, sneeze or talk. These droplets can land in the mouths or noses of people who are nearby | Reference to influenza can be found  since 1650 | Typically characterized by the abrupt onset of constitutional and upper respiratory tract signs and symptoms (e.g., fever, chills, myalgia, headache, malaise, nonproductive cough, sore throat, and rhinitis) | Influenza A viruses are found in many different animals, including ducks, chickens, pigs, whales, horses, seals and cats. Influenza B viruses circulate widely only among humans | Person-to-person transmission through respiratory droplets |
| Measles [14] | Measles morbillivirus | RNA virus | The primary site of infection is alveolar macrophages or dendritic cells. Two to three days after replication in the lung, measles virus spreads to regional lymphoid tissues followed by a systemic infection | The incubation period of measles from exposure to prodrome averages 11 to 12 days | The prodrome lasts 2 to 4 days, with a range of 1 to 7 days.  The measles rash is a maculopapular eruption that usually lasts 5 to 6 days | Measles transmission occurs person-to-person via large respiratory droplets and via airborne transmission of aerosolized droplet nuclei in closed areas | References to  measles can be found as early as the 7th century. | The prodromeis characterized by fever, cough, coryza, and conjunctivitis. Koplik spots, present on mucous membranes, are considered to be unique to measles. Measles rash is a maculopapular eruption. Other symptoms of measles include anorexia and generalized lymphadenopathy. | There is no known animal reservoir | Measles transmission occurs person-to-person via respiratory droplets |
| Brucellosis [15,16] | Brucellosis is can be caused by four different *Brucella* species in humans: *B. suis*, *B. melitensis, B. abortus,* and *B. canis.* *Brucella melitensis* is the most prevalent species | Gram^-^ bacterium | When ingested, *Brucella* organisms are phagocytosed and enter the intestinal submucosa, where they are transported to lymphoid tissue by macrophages. Brucellae are transported into the lymphatic system and may replicate there locally; they also may replicate in the liver, spleen, kidney, breast tissue, or joints, causing both localized and systemic infection | The incubation period of the disease can be highly variable, ranging from 1 week to 2 months, but usually 2–4 weeks | Symptoms frequently last for several months, and chronic infections with brucellosis may last for years | Humans generally acquire the disease through direct contact with infected animals, by eating or drinking contaminated animal products or by inhaling airborne agents. Most cases are caused by ingesting unpasteurized milk or cheese from infected goats or sheep | The disease was described by George Cleghorn in 1751 | Brucellosis typically causes flu-like symptoms, including fever, weakness, malaise and weight loss | Brucellosis is one of the most widespread zoonoses transmitted by animals | Human-to-human transmission is rare |
| COVID-19 [17,18] | SARS-CoV-2 | RNA virus | The virus infects epithelial cells of the lung alveoli by receptor‐mediated endocytosis via the angiotensin‐converting enzyme II (ACE-II) as an entry receptor. High viral loads have been detected in the lower respiratory tract. The main pathogenesis of COVID-19 is severe pneumonia | The incubation period for COVID-19 is thought to extend to 14 days, with a median time of 4-5 days from exposure to symptoms onset | Patients with a mild case of COVID-19 usually recover in one to two weeks. For severe cases, recovery can take six weeks or more | COVID-19 spreads when an infected person breathes out droplets and very small particles that contain the virus. These droplets and particles can be breathed in by other people or land on their eyes, noses, or mouth | Emerged in December 2019 | The signs and symptoms of COVID-19 present at illness onset vary, but over the course of the disease many people with COVID-19 will experience the following: Fever or chills, Cough, Shortness of breath or difficulty breathing, Fatigue, Muscle or body aches, Headache, New loss of taste or smell, Sore throat, Congestion or runny nose, Nausea or vomiting, Diarrhea | Although emerged from animal reservoir, current transmission only sustained by human-to-human transmission | Current transmission only sustained by human-to-human transmission |
| Scarlet fever [19-21] | Scarlet fever is an illness caused by pyrogenic exotoxin-producing S. pyogenes | Gram^+^ bacterium | Group A strep live in the nose and throat. The disease itself is caused by secretion of pyrogenic exotoxins by the infecting *Streptococcus* | It usually takes two to five days for someone exposed to group A strep to become sick | The rash usually persists for about one week | Typically transmission occurs through saliva or nasal secretions from an infected person | Streptococcal diseases have been known for centuries, although their delineation into separate disease entities did not begin to occur until the 16th century | Illness usually begins with a fever and sore throat. There may also be chills, vomiting, or abdominal pain. One or two days after the illness begins, a red rash usually appears. | Humans are the primary reservoir for group A strep | Group A strep infections, including scarlet fever, are most commonly spread through direct person-to-person transmission |
| Western equine encephalitis [22-24] | Western equine encephalitis virus (WEEV) | RNA virus | After the bite of an infected mosquito, viral replication starts in the local lymph nodes. If viral load is high enough, the virus may translocate into the central nervous system resulting in cerebral and meningeal inflammation and necrosis | the incubation period is 5-10 days | Most symptomatic cases will still resolve spontaneously.  Customarily, encephalitis persists for 10 days and then subsides. | The virus is transmitted by mosquitoes | Western equine encephalitis was first identified by Karl Friedrich Meyer, in 1930 following an epizootic outbreak in horses in the San Joaquin Valley in California | The onset is gradual, with mild fever, malaise, headache, photophobia, nausea, vomiting and sore throat, sometimes with meningism and drowsiness. In the minority who progress to encephalitis, the fever and headache increase, with deterioration of conscious state, possibly with flaccid or spastic paralysis | WEEV circulates in more than 75 species of wild birds and some domestic ones. Epizootics in horses acting as amplifying hosts precede human epidemics | Humans are dead-end hosts for the virus |
| Diphtheria [25,26] | *Corynebacterium diphteriae* | Gram^+^ bacterium | The most common sites of diphtheria infection are the pharynx and the tonsils. The organism produces a toxin that inhibits cellular protein synthesis and is responsible for local tissue destruction and membrane formation. The toxin produced at the site of the membrane is absorbed into the bloodstream and then distributed to the tissues of the body. The toxin is responsible for the major complications of myocarditis and neuritis and can also cause low platelet counts (thrombocytopenia) and protein in the urine (proteinuria) | The incubation period for diphtheria is 2 to 5 days, with a range of 1 to 10 days | In untreated people, organisms can be present in discharges and lesions 2 to 6 weeks after infection | Transmission is most often person-to-person through respiratory droplets | Diphtheria was first identified in 1826 by Pierre Bretonneau | Early symptoms include malaise, sore throat, anorexia, and low-grade fever. Within 2 to 3 days, a bluish-white membrane forms and extends, varying in size from covering a small patch on the tonsils to covering most of the soft palate. Patients with severe disease may develop marked edema of the submandibular areas and the anterior neck along with lymphadenopathy, giving a characteristic “bull neck” appearance. If enough toxin is absorbed, the patient can develop severe prostration, pallor, rapid pulse, stupor, and coma | Humans are the reservoir for *C. diphtheriae* | Person-to-person transmission through respiratory droplets |
| Saint Louis encephalitis [27,28] | Saint Louis encephalitis virus | RNA virus | Neuroinvasive disease: encephalitis (inflammation of the brain) or meningitis (inflammation of the membranes that surround the brain and spinal cord) | For people with symptoms, the time from infected mosquito bite to feeling sick ranges from 4 to 14 days | Symptoms typically get worse over a period of several days to a week. Some patients recover after this period. | St. Louis encephalitis (SLE) virus is spread to people through the bite of an infected mosquito | St. Louis encephalitis was first discovered by Joseph F. Bredeck in 1933 following a major outbreak in the city of St. Louis | Symptoms usually start abruptly, with fever, headache, dizziness, nausea, and generalized weakness | Birds that live in urban-suburban areas, such as the house sparrow, pigeon, blue jay, and robin, are common SLE virus hosts | People do not develop high enough levels of the virus in their blood to infect mosquitoes. As a result, people are considered “dead-end” hosts for SLE virus |
| Poliomyelitis [29,30] | Poliovirus | RNA virus | The virus is usually present in nasopharyngeal secretions for 1 to 2 weeks and can be shed in stools for several weeks after infection, even in individuals with minor symptoms or no illness. During intestinal replication, the virus invades local lymphoid tissue and may enter the bloodstream, and then infect cells of the central nervous system. Poliovirus-induced destruction of motor neurons of the anterior horn of the spinal cord and brain stem cells results in distinctive paralysis | The incubation period for nonparalytic poliomyelitis is 3 to 6 days. For the onset of paralysis in paralytic poliomyelitis, the incubation period is usually 7 to 21 days | Abortive poliomyelitis is characterized by a complete recovery in less than a week,  nonparalytic aseptic meningitis has symptoms lasting 2 to 10 days and are followed by complete recovery | Transmission occurs via the fecal-oral or oral-oral routes | The effects of poliomyelitis have been known since prehistory. The first clinical description was provided by the British physician Michael Underwood in 1789 | 70% asymptomatic, 24% abortive poliomyelitis (low fever, sore throat), 1%-5% nonparalytic aseptic meningitis, <1% flaccid paralysis | Humans are the only known reservoir of poliovirus | Person-to-person spread of poliovirus occurs via the fecal-oral or oral-oral routes |
| Typhoid [31-33] | *Salmonella enterica* serotype Typhi | Gram^-^ bacterium | The sequence of events in the pathogenesis of typhoid fever includes gastrointestinal infection, systemic involvement, and chronic carrier state | The incubation period of typhoid infection is 6–30 days | Untreated the disease can last for 1 month | Typhoid is acquired through consumption of water or food contaminated by feces of an acutely infected or convalescent person or a chronic, asymptomatic carrier | In 1880, German pathologist Karl Joseph Eberth discovered the microbe that causes typhoid fever | Headache, fever, malaise, and anorexia are nearly universal, and abdominal pain, diarrhea, or constipation are common. Vomiting and diarrhea are more common in children compared with adults. People can also have fatigue, myalgias, dry cough, and sore throat | No animal reservoirs have been identified | Person-to-person transmission through the fecal-oral route |
| SARS [34-36] | SARS-CoV | RNA virus | SARS-CoV produces the infection of lower respiratory tract, most patients developing pneumonia | The incubation period of SARS is usually 2-7 days but may be as long as 10 days | The average duration of onset of symptoms to hospital discharge in patients who survived is 33 days | SARS is an airborne virus thought to be transmitted most readily by respiratory droplets produced when an infected person coughs or sneezes | Emerged at the end of 2002 | The first symptom of the illness is generally fever associated with chills and rigors. It may also be accompanied by other symptoms including headache, malaise, and muscle pain. After 3-7 days, a lower respiratory phase begins with the onset of a dry, non-productive cough or dyspnoea that may be accompanied by hypoxemia. In 10–20% of cases, the respiratory illness is severe enough to require intubation and mechanical ventilation | The 2003 outbreak was sustained by human-to-human transmission although the virus emerged from an animal reservoir | The primary way that SARS appears to spread is by close person-to-person contact |
| Japanese encephalitis [37,38] | Japanese encephalitis virus (JEV) | RNA Virus | Infection of the central nervous system producing encephalitis | The incubation period is between 4-14 days | Average duration of symptoms was 7-8 days in children, mean duration of stay in hospital was 3 weeks, but follow up during the following 12 months showed persistent behavioural and neurological deficit | JEV is transmitted to humans through bites from infected mosquitoes | The first case of Japanese encephalitis viral disease was documented in 1871 in Japan | Severe disease is characterized by rapid onset of high fever, headache, neck stiffness, disorientation, coma, seizures, spastic paralysis and ultimately death | The virus exists in a transmission cycle between mosquitoes, pigs and/or water birds (enzootic cycle) | Humans, once infected, do not develop sufficient viraemia to infect feeding mosquitoes |
| MERS [39-41] | MERS-CoV | RNA virus | During the course of the infection, MERS-CoV is mainly detected in the lower respiratory tract | The median incubation period for secondary cases associated with limited human-to-human transmission is approximately 5 days (range 2-14 days) | From 1 to 4 weeks depending on the severity of the disease; median time from onset to death is approximately 12 days. In one series of 12 ICU patients, the median duration of mechanical ventilation was 16 days, and median ICU length of stay was 30 days | MERS-CoV, like other coronaviruses, likely spreads from an infected person’s respiratory secretions, such as through coughing | Emerged in 2012 | A wide clinical spectrum of MERS-CoV infection has been reported ranging from asymptomatic infection to acute upper respiratory illness, and rapidly progressive pneumonitis, respiratory failure, septic shock and multi-organ failure resulting in death | MERS-CoV) is a virus transferred to humans from infected dromedary camels | Human-to-human transmission is possible, but only a few such transmissions have been found among family members living in the same household. In health care settings, however, human-to-human transmission appears to be more frequent |
| Typhus [42-47] | *Rickettsia prowazekii* | Gram^-^ bacterium | Following transmission, rickettsia are ingested by macrophages and polymorphonuclear cells. The major pathology is caused by a vasculitis and its complications. This process may result in occlusion of blood vessels and initiates inflammatory response. This vasculitic process causes destruction of the endothelial cells and leakage of the blood leading to volume depletion with subsequent hypovolemia and decreased tissue perfusion and, possibly organ failure | The incubation period of epidemic louse-borne typhus is typically between 10 and 14 days | In uncomplicated epidemic typhus, fever usually resolves after 2 weeks of illness if untreated | Epidemic typhus is spread to people through contact with infected body lice | Paleomicrobiology enabled the identification of the first outbreak of epidemic typhus in the 18th century in the city of Douai, France | Fever and chills, headache, rapid breathing, myalgia, rash, cough, nausea, vomiting, confusion | In the United States, rare cases of epidemic typhus, called sylvatic typhus, can occur. These cases occur when people are exposed to flying squirrels and their nests | People become infected with *R. prowazekii* when they come into contact with the feces or crushed bodies of infected lice via cut or injured skin. |
| Smallpox [48-50] | Variola Major (accounting for over 85% of all cases during the smallpox era) | DNA virus | The smallpox virus commonly enters the body through the upper respiratory tract. The infection commonly progresses by entering  macrophages and spreading to lymph nodes and producing exuberant secondary viraemia.  During secondary viraemia the virus infects mucous cells of the pharynx and mouth, and endothelium of the capillaries of the dermis, causing skin lesions. Other organs with high viral loads include spleen, liver, bone marrow and kidneys | Infection with variola virus begins with an incubation period usually lasting between 10 to 14 days (range 7 to 19 days) | 3-4 weeks | Infected people spread the virus when they coughed or sneezed and droplets from their nose or mouth spread to other people. Transmission via contact with material from the smallpox pustules or crusted scabs can also occur. Scabs are much less infectious than respiratory secretions | The finding of smallpox-like rashes on Egyptian mummies suggests that smallpox has existed for at least 3,000 years | Prodrome: fever, malaise, prostration, headache, backache, vomiting, severe abdominal pain, chills, anorexia, pharyngitis  eruptive stage: rash lesions | There are no known animal reservoirs of smallpox | Human-to-human transmission of variola virus occurs by inhalation of large, virus-containing airborne droplets of saliva from an infected person |
| Yellow fever [51-53] | Yellow fever virus | RNA virus | After infection the virus first replicates locally, followed by transportation to the rest of the body via the lymphatic system.  Following systemic lymphatic infection the virus proceeds to establish itself throughout organ systems, including the heart, kidneys, adrenal glands, and the parenchyma of the liver; high viral loads are also present in the blood | The incubation period is typically 3–6 days | In most cases, symptoms disappear after 3 to 4 days; half of the patients who enter the toxic phase die within 7 - 10 days | Vectorborne transmission of YF virus occurs via the bite of an infected mosquito | One of the most  feared infectious diseases from the 15th to 19th centuries | Most patients improve after the initial presentation. After a brief remission of up to 24 hours, approximately 12% of those infected progress to a more serious form of the disease, characterized by jaundice, hemorrhagic symptoms, and eventually shock and multisystem organ failure | Nonhuman and human primates are the main reservoirs of the virus | Anthroponotic (human-to-vector-to-human) transmission |
| Cholera [54-56] | *Vibrio cholera* | Gram^-^ bacterium | Cholera is an acute diarrhoeal infection | It usually takes 2-3 days for symptoms to appear after a person ingests cholera bacteria, but the time can range from a few hours to 5 days | In the majority of cases, cholera is characterized by  acute, profuse watery diarrhoea of 1 or a few days’ duration.  In its extreme manifestation, cholera is one of  the most rapidly fatal infectious illnesses known. Within  3–4 hours of onset of symptoms, a previously healthy  person may become severely dehydrated and if not  treated may die within 24 hours | A person can get cholera by drinking water or eating food contaminated with the bacteria | During the 19th century, cholera spread across the world from its original reservoir in the Ganges delta in India | Among people who develop symptoms, the majority have mild or moderate symptoms, while a minority develop acute watery diarrhoea with severe dehydration | *Vibrio cholera* has an environmental reservoir in brackish or saltwater | Human-to-human transmission through the fecal-oral route |
| Lassa fever [57,58] | Lassa mammarenavirus | RNA virus | Lassa fever may potentially infect all organs, but the liver and auditory sensorineural system are commonly involved | The incubation period of Lassa fever ranges from 6–21 days | Lassa fever is an acute viral haemorrhagic illness of 2-21 days duration | Transmission of Lassa virus to humans occurs most commonly through ingestion or inhalation. Mastomys rodents shed the virus in urine and droppings and direct contact with these materials, through touching soiled objects, eating contaminated food, or exposure to open cuts or sores, can lead to infection.  Contact with the virus may also occur when a person inhales tiny particles in the air contaminated with infected rodent excretions. Lassa virus may also be spread between humans through direct contact with the blood, urine, faeces, or other bodily secretions of an infected person | Though first described in the 1950s, the virus causing Lassa disease was not identified until 1969 | Mild symptoms include slight fever, general malaise and weakness, and headache. In 20% of infected individuals, however, disease may progress to more serious symptoms including hemorrhaging (in gums, eyes, or nose, as examples), respiratory distress, repeated vomiting, facial swelling, pain in the chest, back, and abdomen, and shock | The animal reservoir, or host, of Lassa virus is a rodent of the genus Mastomys | Lassa virus may be spread between humans through direct contact with the blood, urine, faeces, or other bodily secretions of an infected person. Person-to-person transmission occurs in both community and health-care settings. Sexual transmission of Lassa virus has been reported |
| AIDS [59,60] | Human immunodeficiency virus 1 | RNA virus | HIV causes an acquired immunodeficiency by depleting CD4+ T helper lymphocytes | Some people have flu-like symptoms within 2 to 4 weeks after infection | HIV produces AIDS several years after the infection | Transmission occurs through contact with body fluids | From the 1920s Kinshasa was the focus of early HIV-1 transmission | Acute stage: flu-like symptoms. Chronic stage: immune suppression and opportunistic infections | No, although emerged from an animal reservoir | Transmission during sexual intercourses or contact with infected blood. Mother-to-child transmission is also possible |
| Pulmonary tuberculosis [61-64] | *Mycobacterium tuberculosis* | Bacterium | Pulmonary tuberculosis refers to the infection of lungs. Extrapulmonary tuberculosis occurs when other organs are infected | Typically several months | The duration of tuberculosis from onset to cure or death is approximately 3 years | TB bacteria spread through the air from one person to another | Known since ancient Greece and Rome | Cough lasting 3 weeks or longer, pain in the chest, coughing up blood, fatigue, weight loss, lung damage | Humans are the reservoir hosts of *M. tuberculosis* | TB bacteria spread through the air from one person to another |
| Meningococcal meningitis [65-68] | *Neisseria meningitis* | Gram^-^ bacterium | The bacteria attach  to and multiply in the mucosal cells of the nasopharynx and  oropharynx and, in a small proportion (much less than 1%) of  persons, penetrate the mucosal cells and enter the bloodstream.  The bacteria can then spread through the blood to cause  systemic disease and cross the blood-brain barrier into the  cerebrospinal fluid (CSF) to cause meningitis | The incubation period is 3-4 days (range 1-10 days) | Median (interquartile range) duration of hospital admission was 10 (8–13) days | Meningococci are transmitted person-to-person by respiratory  droplets or secretions from persons with asymptomatic  colonization or meningococcal disease | Meningococcal  disease was first described in 1805 when an outbreak swept  through Geneva, Switzerland. The causative agent, Neisseria men-  ingitidis (the meningococcus), was identified in 1887 | Meningitis, ever, headache, and stiff  neck, often accompanied by other symptoms, such as nausea,  vomiting, photophobia (eye sensitivity to light), and altered  mental status, Meningococcal septicaemia, bacteremic pneumonia | *N. meningitidis* only infects humans; there is no animal reservoir | Meningococci are transmitted person-to-person by respiratory  droplets |
| Ebola [69-71] | Ebolavirus | RNA virus | The Ebola virus infects the mononuclear phagocyte system, but also other cells such as hepatocytes, spongiocytes, fibroblasts and endothelial cells, inducing tissue necrosis and disrupting the hematological and coagulation systems | The incubation period for Ebola, from exposure to when signs or symptoms appear, can be anywhere from 2 to 21 days. The average is 8 to 10 days | Survivors start to recover about 2 weeks after onset of disease, but some symptoms (eg, arthralgia) can persist during 6 weeks | Ebola is introduced into the human population through close contact with the blood, secretions, organs or other bodily fluids of infected animals. Ebola then spreads through human-to-human transmission via direct contact with blood or body fluids of a person who is sick with or has died from Ebola | EVD first appeared in 1976 in 2 simultaneous outbreaks, one in what is now Nzara, South Sudan, and the other in Yambuku, DRC | fever, fatigue, myalgia, headache, vomiting, diarrhea, rash, impaired kidney and liver function, internal and external bleeding | It is thought that fruit bats of the Pteropodidae family are natural Ebola virus hosts | Ebola spreads through human-to-human transmission via direct contact with blood or body fluids of a person who is sick with or has died from Ebola |
| Plague [72-74] | *Yersinia pestis* | Gram^-^ bacterium | When bubonic plague is left untreated, plague bacteria can invade the bloodstream. When plague bacteria multiply in the bloodstream, they spread rapidly throughout the body and cause a severe and often fatal condition called septicemic plague. Untreated bubonic plague can also progress into an infection of the lungs, causing pneumonic plague | A person usually becomes ill with bubonic plague 2 to 8 days after being infected. The incubation period of septicemic plague is poorly defined but likely occurs within days of exposure. A person exposed to *Yersinia pestis* through the air would usually become ill in just 1 to 3 days | Pneumonic plague fatal within 3 days;  recovery from bubonic plague in treated patients after 10-14 days (longer for more severe infections) | Plague is transmitted between animals through fleas. Humans can be infected through: the bite of infected vector fleas, unprotected contact with infectious bodily fluids or contaminated materials, the inhalation of respiratory droplets from a patient with pneumonic plague | Known since ancient Greece | Bubonic plague: fever, headache, chills, weakness, painful lymph nodes;  septicemic plague: fever, chills, extreme weakness, abdominal pain, shock, and possibly bleeding into the skin and other organs;  pneumonic plague: fever, headache, weakness, and a rapidly developing pneumonia with shortness of breath, chest pain, cough, and sometimes bloody or watery mucous | *Yersinia pestis* is found in small mammals and their fleas | Human-to-human transmission occurs through the inhalation of respiratory droplets from a patient with pneumonic plague |

1. <https://www.who.int/news-room/fact-sheets/detail/campylobacter>
2. Sheppard SK, Maiden MCJ. The evolution of Campylobacter jejuni and Campylobacter coli. Cold Spring Harb Perspect Biol 2015; 7, a018119.
3. <https://www.who.int/news-room/fact-sheets/detail/dengue-and-severe-dengue>
4. Gubler DJ. Dengue/dengue haemorragic fever: history and current status. Novartis Found Symp 2006; 277, 3-16.
5. https://www.wikidoc.org/index.php/Dengue_fever
6. <https://www.who.int/news-room/fact-sheets/detail/hepatitis-a>
7. <https://www.cdc.gov/vaccines/pubs/pinkbook/hepa.html>
8. <https://www.cdc.gov/vaccines/pubs/pinkbook/pert.html>
9. <https://www.cdc.gov/salmonella/index.html>
10. https://www.cdc.gov/training/SIC_CaseStudy/Infection_Salmonella_ptversion.pdf
11. <https://www.cdc.gov/flu/about/keyfacts.htm>
12. <https://www.cdc.gov/flu/professionals/acip/clinical.htm>
13. Potter CW. A history of influenza. J Appl Microbiol 2001; 91, 572-579.
14. <https://www.cdc.gov/vaccines/pubs/pinkbook/downloads/meas.pdf>
15. <https://www.who.int/news-room/fact-sheets/detail/brucellosis>
16. Hayoun MA, Muco E, Shorman M. Brucellosis. In: StatPearls [Internet]. Treasure Island (FL): StatPearls Publishing; 2022.
17. <https://www.cdc.gov/coronavirus/2019-ncov/hcp/clinical-guidance-management-patients.html>
18. <https://www.hopkinsmedicine.org/health/conditions-and-diseases/coronavirus/diagnosed-with-covid-19-what-to-expect>
19. <https://www.cdc.gov/groupastrep/diseases-public/scarlet-fever.html>
20. <https://www.cdc.gov/groupastrep/diseases-hcp/scarlet-fever.html>
21. Ferretti J, Köhler W. History of Streptococcal Research. In Ferretti JJ, Stevens DL, Fischetti VA (Eds) Streptococcus pyogenes. Basic biology to Clinical Manifestations. The University of Oklahoma Health Sciences Center (2016).
22. Simon LV, Coffey R, Fischer MA. Western Equine Encephalitis. In: StatPearls [Internet]. Treasure Island (FL): StatPearls Publishing; 2022.
23. Young PR, Ng LFP, Hall RA, Smith DW, Johansen CA. Arboviral infections. Manson's Tropical Infectious Diseases (Twenty-Third Edition), 2014, Pages 129-161.
24. Craighead JE. Neurotropic arthropod-transmitted viruses. In: Pathology and Pathogenesis of human viral disease. Academic Press, 2000, pages 343-356.
25. <https://www.cdc.gov/vaccines/pubs/pinkbook/dip.html>
26. <https://www.who.int/immunization/monitoring_surveillance/burden/vpd/WHO_SurveillanceVaccinePreventable_04_Diphtheria_R2.pdf>
27. <https://www.cdc.gov/sle/index.html>
28. Editorial. Encephalitis in St. Louis 1933. Am J Public Health Nations Health 1933; 23, 1058-1060.
29. <https://www.cdc.gov/polio/index.htm>
30. <https://www.cdc.gov/vaccines/pubs/pinkbook/polio.html#pathogenesis>
31. <https://wwwnc.cdc.gov/travel/yellowbook/2020/travel-related-infectious-diseases/typhoid-and-paratyphoid-fever>
32. Adler R, Mara E. Typhoid fever: a history. McFarland & Company, Jefferson, North Carolina (2016).
33. https://www.wikidoc.org/index.php/Typhoid_fever_overview
34. <https://www.who.int/health-topics/severe-acute-respiratory-syndrome#tab=tab_2>
35. <https://www.cdc.gov/sars/about/faq.html>
36. Feng D, Jia N, Fang L-Q, Richardus JH, Han X-N, Cao W-C, et al. Duration of symptom onset to hospital admission and admission to discharge or death in SARS in mainland China: a descriptive study. Trop Med Int Health 2009; 14, 28-35.
37. <https://www.who.int/news-room/fact-sheets/detail/japanese-encephalitis>
38. Singh A, Mehta A, Kushwaha KP, Pandey AK, Mittal M, Sharma B, et al. Minocycline trial in Japanese encephalitis: a double blind, randomized placebo study. Int J Pediatr Res 2016; 3, 371-377.
39. <https://www.cdc.gov/coronavirus/mers/clinical-features.html>
40. Virlogeux V, Park M, Wu JT, Cowling BJ. Association between severity of MERS-CoV infection and incubation period. Emerg Infect Dis 2016; 22, 526-528.
41. <https://www.who.int/health-topics/middle-east-respiratory-syndrome-coronavirus-mers#tab=tab_2>
42. <https://www.cdc.gov/typhus/epidemic/index.html>
43. <https://www.ecdc.europa.eu/en/epidemic-louse-borne-typhus/facts>
44. <https://www.cdc.gov/typhus/murine/faq.html>
45. Angelakis E, Raoult D. Rickettsia and Rickettsia-like organisms. In: Cohen J, Infectious Diseases (fourth edition), 2017, pages 1666-1675. Elsevier.
46. Angelakis E, Bechah Y, Raoult D. The history of epidemic typhus. Microbiol Spectr 2016; 4.
47. https://www.wikidoc.org/index.php/Typhus_pathophysiology
48. <https://www.cdc.gov/smallpox/clinicians/clinical-disease.html>
49. <https://www.cdc.gov/smallpox/history/history.html>
50. <https://www.cdc.gov/smallpox/symptoms/index.html>
51. <https://wwwnc.cdc.gov/travel/yellowbook/2020/travel-related-infectious-diseases/yellow-fever>
52. <https://www.who.int/news-room/fact-sheets/detail/yellow-fever>
53. Bryant JE, Holmes EC, Barrett AD. Out of Africa : A molecular perspective on the introduction of yellow fever virus into the Americas. PLoS Pathog 2007; 3: e75.
54. <https://www.who.int/news-room/fact-sheets/detail/cholera>
55. <https://www.cdc.gov/cholera/general/index.html#four>
56. <https://www.who.int/wer/2010/wer8513.pdf>
57. <https://www.cdc.gov/vhf/lassa/index.html>
58. <https://www.who.int/news-room/fact-sheets/detail/lassa-fever>
59. <https://www.cdc.gov/hiv/basics/whatishiv.html>
60. Faria NR, Rambaut A, Suchard MA, Baele G, Bedford T, Ward MJ, et al. The early spread and epidemic ignition of HIV-1 in human populations. Science 2014; 346, 56-61.
61. <https://www.cdc.gov/tb/topic/basics/signsandsymptoms.htm>
62. Behr MA, Edelstein PH, Ramakrishnan L. Revisiting the timetable of tuberculosis BMJ 2018 :362:k2738.
63. Tiemersma EW, van der Werf MJ, Borgdorff MW, Williams BG, Nagelkerke NJD. Natural history of tuberculosis: duration and fatality of untreated pulmonary tuberculosis in HIV negative patients: a systematic review. PLoS ONE 2011; 6, e17601.
64. <https://www.cdc.gov/tb/worldtbday/history.htm>
65. Tyler KL. A history of bacterial meningitis. Handb Clin Neurol 2010; 95, 417-433.
66. <http://apps.who.int/iris/bitstream/handle/10665/232232/WER7833_294-296.PDF?sequence=1>
67. <https://www.cdc.gov/vaccines/pubs/pinkbook/downloads/mening.pdf>
68. Stoof SP, Rodenburg GD, Knol MJ, Rümke LW, Bovenkerk S, Berbers GAM, et al. Disease burden of invasive meningococcal disease in the Netherlands between june 1999 and june 2011: a subjective role for serogroup and clonal complex. Clin Infect Dis 2015; 61, 1281-1292.
69. <https://www.who.int/news-room/fact-sheets/detail/ebola-virus-disease>
70. <https://www.cdc.gov/dotw/ebola/index.html>
71. Bwaka MA, Bonnet MJ, Calain P, Colebunders R, De Roo A, Guimard Y, et al. Ebola hemorrhagic fever in Kikwit, Democratic Republic of the Congo: clinical observations in 103 patients. J Infect Dis 1999; 179, S1-7.
72. <https://www.cdc.gov/plague/faq/index.html#incubation>
73. <https://www.who.int/news-room/fact-sheets/detail/plague>
74. <https://www.cdc.gov/plague/healthcare/clinicians.html>
