# Supplementary material for: Age-dependent virulence of human pathogens
Source: PLoS Pathog. 2022 Sep 22;18(9):e1010866. doi: 10.1371/journal.ppat.1010866 (PMC9531802; doi:10.1371/journal.ppat.1010866)
Supplement: S2 Table — We report the -2 Log Likelihood, AIC, BIC, Pearson Statistics, number of parameters (k), the overdispersion parameter (Pearson Statistic/(N-k), and the ΔBIC. N = 873 observations. We ran 19 competitive finite mixture models. The model with the lowest BIC value is highlighted in green. (DOCX) [file ppat.1010866.s002.docx]

S2 Table. Model comparison on the effect of duration of symptoms/illness, incubation period, local/systemic infection on age specific CFR for 28 human infectious diseases. We report the -2 Log Likelihood, AIC, BIC, Pearson Statistics, number of parameters (k), the overdispersion parameter (Pearson Statistic/(N-k), and the ΔBIC. N = 873 observations. We ran 19 competitive finite mixture models. The model with the lowest BIC value is highlighted in green.

|  | **-2 Log Likelihood** | **AIC** | **BIC** | **Pearson Statistic** | **k** | **Pearson Statistic/(N-k)** | **ΔBIC** |
| --- | --- | --- | --- | --- | --- | --- | --- |
| *Main factors* |  |  |  |  |  |  |  |
| 1. Intercept | 8505.2 | 8509.2 | 8518.7 | 883.4 | 2 | 1.014 | 270.2 |
| 1. Age | 8478.4 | 8484.4 | 8498.7 | 870.9 | 3 | 1.001 | 250.2 |
| 1. Age + Age² | 8473.9 | 8481.9 | 8500.9 | 875.1 | 4 | 1.007 | 252.4 |
| 1. Age + Age² + Date + Intertropical | 8232.7 | 8244.7 | 8273.3 | 847.6 | 6 | 0.978 | 24.8 |
| 1. Age + Age² + Date + Intertropical + A + B + C | 8195.1 | 8213.1 | 8256 | 830.8 | 9 | 0.962 | 7.5 |
|  |  |  |  |  |  |  |  |
| *Interactions between Age and A,B,C* |  |  |  |  |  |  |  |
| 1. 4 + Age * A + Age * B + Age * C | 8173.7 | 8197.7 | 8255 | 840 | 12 | 0.976 | 6.5 |
| 1. 4 + Age * A + Age * B | 8178.6 | 8200.6 | 8253.1 | 837.9 | 11 | 0.972 | 4.6 |
| 1. 4 + Age * A + Age * C | 8177.8 | 8199.8 | 8252.2 | 841 | 11 | 0.976 | 3.7 |
| 1. 4 + Age * B + Age * C | 8181.3 | 8203.3 | 8255.8 | 835.6 | 11 | 0.969 | 7.3 |
| 1. 4 + Age * A | 8180 | 8200 | 8248.5 | 839.5 | 10 | 0.973 | 0 |
| 1. 4 + Age * B | 8183.8 | 8203.8 | 8251.5 | 833.3 | 10 | 0.966 | 3 |
| 1. 4 + Age * C | 8195 | 8215 | 8262.8 | 830.7 | 10 | 0.963 | 14.3 |
|  |  |  |  |  |  |  |  |
| *Interactions between Age² and A,B,C* |  |  |  |  |  |  |  |
| 1. 5 + Age² * A + Age² * B + Age² * C | 8174.1 | 8204.1 | 8275.7 | 853.6 | 15 | 0.995 | 27.2 |
| 1. 5 + Age² * A + Age² * B | 8201.5 | 8229.5 | 8296.3 | 856.6 | 14 | 0.997 | 47.8 |
| 1. 5 + Age² * A + Age² * C | 8172.4 | 8200.4 | 8267.2 | 836.3 | 14 | 0.974 | 18.7 |
| 1. 5 + Age² * B + Age² * C | 8171.5 | 8199.5 | 8266.3 | 838.1 | 14 | 0.976 | 17.8 |
| 1. 5 + Age² * A | 8173.7 | 8199.7 | 8261.7 | 839.7 | 13 | 0.976 | 13.2 |
| 1. 5 + Age² * B | 8171.4 | 8197.4 | 8259.4 | 840.1 | 13 | 0.977 | 10.9 |
| 1. 5 + Age² * C | 8172.5 | 8198.5 | 8260.5 | 836.4 | 13 | 0.973 | 12 |

A = duration of symptoms/illness; B = incubation period; C = local/systemic infection
