# Supplementary material for: Age-dependent virulence of human pathogens
Source: PLoS Pathog. 2022 Sep 22;18(9):e1010866. doi: 10.1371/journal.ppat.1010866 (PMC9531802; doi:10.1371/journal.ppat.1010866)
Supplement: S3 Table — We report the -2 Log Likelihood, AIC, BIC, Pearson Statistics, number of parameters (k), the overdispersion parameter (Pearson Statistic/(N-k), and the ΔBIC. N = 873 observations. We ran 35 competitive finite mixture models. The model with the lowest BIC value is highlighted in green. (DOCX) [file ppat.1010866.s003.docx]

S3 Table. Model comparison on the effect of pathogen type, length of human-pathogen association, animal reservoir and human-to-human transmission on age specific CFR for 28 human infectious diseases. We report the -2 Log Likelihood, AIC, BIC, Pearson Statistics, number of parameters (k), the overdispersion parameter (Pearson Statistic/(N-k), and the ΔBIC. N = 873 observations. We ran 35 competitive finite mixture models. The model with the lowest BIC value is highlighted in green.

|  | **-2 Log Likelihood** | **AIC** | **BIC** | **Pearson Statistic** | **k** | **Pearson Statistic/(N-k)** | **ΔBIC** |
| --- | --- | --- | --- | --- | --- | --- | --- |
| *Main factors* |  |  |  |  |  |  |  |
| 1. Intercept | 8505.2 | 8509.2 | 8518.7 | 883.4 | 2 | 1.014 | 347.4 |
| 1. Age | 8478.4 | 8484.4 | 8498.7 | 870.9 | 3 | 1.001 | 327.4 |
| 1. Age + Age² | 8473.9 | 8481.9 | 8500.9 | 875.1 | 4 | 1.007 | 329.6 |
| 1. Age + Age² + Date + Intertropical | 8232.7 | 8244.7 | 8273.3 | 847.6 | 6 | 0.978 | 102 |
| 1. Age + Age² + Date + Intertropical + A + B + C + D | 8111.3 | 8131.3 | 8179 | 973.4 | 10 | 1.128 | 7.7 |
|  |  |  |  |  |  |  |  |
| *Interactions between Age and A,B,C,D* |  |  |  |  |  |  |  |
| 1. 4 + Age * A + Age * B + Age * C + Age * D | 8086 | 8114 | 8180.8 | 1041.5 | 14 | 1.212 | 9.5 |
| 1. 4 + Age * A + Age * B + Age * C | 8088 | 8114 | 8176 | 1019.1 | 13 | 1.185 | 4.7 |
| 1. 4 + Age * A + Age * B + Age * D | 8086.5 | 8112.5 | 8174.5 | 1049.5 | 13 | 1.22 | 3.2 |
| 1. 4 + Age * A + Age * C + Age * D | 8105.3 | 8131.3 | 8193.3 | 981.9 | 13 | 1.142 | 22 |
| 1. 4 + Age * B + Age * C + Age * D | 8100.7 | 8126.7 | 8188.7 | 975.1 | 13 | 1.134 | 17.4 |
| 1. 4 + Age * A + Age * B | 8090 | 8114 | 8171.3 | 1027.2 | 12 | 1.193 | 0 |
| 1. 4 + Age * A + Age * C | 8105.4 | 8129.4 | 8186.6 | 982.4 | 12 | 1.141 | 15.3 |
| 1. 4 + Age * A + Age * D | 8107.9 | 8131.9 | 8189.2 | 986.7 | 12 | 1.146 | 17.9 |
| 1. 4 + Age * B + Age * C | 8101.2 | 8125.2 | 8182.5 | 971.3 | 12 | 1.128 | 11.2 |
| 1. 4 + Age * B + Age * D | 8102.9 | 8126.9 | 8184.1 | 982.6 | 12 | 1.141 | 12.8 |
| 1. 4 + Age * C + Age * D | 8107.7 | 8131.7 | 8189 | 969.1 | 12 | 1.126 | 17.7 |
| 1. 4 + Age * A | 8108.1 | 8130.1 | 8182.6 | 986.9 | 11 | 1.145 | 11.3 |
| 1. 4 + Age * B | 8105 | 8127 | 8179.5 | 978.2 | 11 | 1.135 | 8.2 |
| 1. 4 + Age * C | 8107.7 | 8129.7 | 8182.2 | 969.4 | 11 | 1.125 | 10.9 |
| 1. 4 + Age * D | 8111 | 8133 | 8185.4 | 972.9 | 11 | 1.129 | 14.1 |
|  |  |  |  |  |  |  |  |
| *Interactions between Age² and A,B,C,D* |  |  |  |  |  |  |  |
| 1. 5 + Age² * A + Age² * B + Age² * C + Age² * D | 8084.3 | 8120.3 | 8206.2 | 1047.9 | 18 | 1.226 | 34.9 |
| 1. 5 + Age² * A + Age² * B + Age² * C | 8085.2 | 8119.2 | 8200.3 | 1040.4 | 17 | 1.215 | 29 |
| 1. 5 + Age² * A + Age² * B + Age² * D | 8084.8 | 8118.8 | 8199.9 | 1045.3 | 17 | 1.221 | 28.6 |
| 1. 5 + Age² * A + Age² * C + Age² * D | 8084.5 | 8118.5 | 8199.6 | 1051 | 17 | 1.228 | 28.3 |
| 1. 5 + Age² * B + Age² * C + Age² * D | 8084.3 | 8118.3 | 8199.5 | 1048.2 | 17 | 1.225 | 28.2 |
| 1. 5 + Age² * A + Age² * B | 8085.3 | 8117.3 | 8193.6 | 1040.1 | 16 | 1.214 | 22.3 |
| 1. 5 + Age² * A + Age² * C | 8085.9 | 8117.9 | 8194.2 | 1041.2 | 16 | 1.215 | 22.9 |
| 1. 5 + Age² * A + Age² * D | 8085.1 | 8117.1 | 8193.5 | 1048.6 | 16 | 1.224 | 22.2 |
| 1. 5 + Age² * B + Age² * C | 8085.3 | 8117.3 | 8193.6 | 1040.5 | 16 | 1.214 | 22.3 |
| 1. 5 + Age² * B + Age² * D | 8084.8 | 8116.8 | 8193.2 | 1045.9 | 16 | 1.22 | 21.9 |
| 1. 5 + Age² * C + Age² * D | 8084.5 | 8116.5 | 8192.9 | 1050.9 | 16 | 1.226 | 21.6 |
| 1. 5 + Age² * A | 8086 | 8116 | 8187.6 | 1041.5 | 15 | 1.214 | 16.3 |
| 1. 5 + Age² * B | 8085.4 | 8115.4 | 8187 | 1040.5 | 15 | 1.213 | 15.7 |
| 1. 5 + Age² * C | 8085.9 | 8115.9 | 8187.5 | 1041.5 | 15 | 1.214 | 16.2 |
| 1. 5 + Age² * D | 8085.1 | 8115.7 | 8186.7 | 1048.4 | 15 | 1.222 | 15.4 |

A = pathogen type; B = length of human-pathogen association; C = animal reservoir; D = human-to-human transmission
