# Supplementary material for: Age-dependent virulence of human pathogens
Source: PLoS Pathog. 2022 Sep 22;18(9):e1010866. doi: 10.1371/journal.ppat.1010866 (PMC9531802; doi:10.1371/journal.ppat.1010866)
Supplement: S4 Table — We report the -2 Log Likelihood, AIC, BIC, Pearson Statistics, number of parameters (k), the overdispersion parameter (Pearson Statistic/(N-k), and the ΔBIC. N = 443 observations. We ran 19 competitive finite mixture models. The model with the lowest BIC value is highlighted in green. (DOCX) [file ppat.1010866.s004.docx]

S4 Table. Model comparison on the effect of length of human-pathogen association, animal reservoir and human-to-human transmission on age specific CFR for the restricted dataset including only viral diseases. We report the -2 Log Likelihood, AIC, BIC, Pearson Statistics, number of parameters (k), the overdispersion parameter (Pearson Statistic/(N-k), and the ΔBIC. N = 443 observations. We ran 19 competitive finite mixture models. The model with the lowest BIC value is highlighted in green.

|  | **-2 Log Likelihood** | **AIC** | **BIC** | **Pearson Statistic** | **k** | **Pearson Statistic/(N-k)** | **ΔBIC** |
| --- | --- | --- | --- | --- | --- | --- | --- |
| *Main factors* |  |  |  |  |  |  |  |
| 1. Intercept | 4148.2 | 4152.2 | 4160.4 | 464.6 | 2 | 1.053 | 71.2 |
| 1. Age | 4126 | 4132 | 4144.2 | 465.2 | 3 | 1.057 | 55 |
| 1. Age + Age² | 4118.6 | 4126.6 | 4143 | 480.3 | 4 | 1.094 | 53.8 |
| 1. Age + Age² + Date + Intertropical | 4083.8 | 4095.8 | 4120.3 | 414.6 | 6 | 0.949 | 31.1 |
| 1. Age + Age² + Date + Intertropical + A + B + C | 4043.4 | 4061.4 | 4098.2 | 444.5 | 9 | 1.024 | 9 |
|  |  |  |  |  |  |  |  |
| *Interactions between Age and A,B,C* |  |  |  |  |  |  |  |
| 1. 4 + Age * A + Age * B + Age * C | 4027.3 | 4051.3 | 4100.5 | 477.6 | 12 | 1.108 | 11.3 |
| 1. 4 + Age * A + Age * B | 4028 | 4050 | 4095.1 | 465.6 | 11 | 1.078 | 5.9 |
| 1. 4 + Age * A + Age * C | 4027.3 | 4049.3 | 4094.4 | 477.2 | 11 | 1.105 | 5.2 |
| 1. 4 + Age * B + Age * C | 4041.7 | 4063.7 | 4108.7 | 444.3 | 11 | 1.028 | 19.5 |
| 1. 4 + Age * A | 4028.8 | 4048.2 | 4089.2 | 467.7 | 10 | 1.080 | 0 |
| 1. 4 + Age * B | 4043 | 4063 | 4104 | 443.9 | 10 | 1.025 | 14.8 |
| 1. 4 + Age * C | 4042.8 | 4062.8 | 4103.7 | 445.1 | 10 | 1.028 | 14.5 |
|  |  |  |  |  |  |  |  |
| *Interactions between Age² and A,B,C* |  |  |  |  |  |  |  |
| 1. 5 + Age² * A + Age² * B + Age² * C | 4025.8 | 4055.8 | 4117.2 | 480.1 | 15 | 1.122 | 28 |
| 1. 5 + Age² * A + Age² * B | 4026.1 | 4054.1 | 4111.4 | 476.2 | 14 | 1.110 | 22.2 |
| 1. 5 + Age² * A + Age² * C | 4025.9 | 4053.9 | 4111.2 | 481.3 | 14 | 1.122 | 22 |
| 1. 5 + Age² * B + Age² * C | 4025.9 | 4053.9 | 4111.2 | 482.7 | 14 | 1.125 | 22 |
| 1. 5 + Age² * A | 4026.8 | 4052.8 | 4106 | 475.5 | 13 | 1.106 | 16.8 |
| 1. 5 + Age² * B | 4026.8 | 4052.8 | 4106 | 477.7 | 13 | 1.111 | 16.8 |
| 1. 5 + Age² * C | 4026 | 4052 | 4105.2 | 483.4 | 13 | 1.124 | 16 |

A = Length of association, B = animal reservoir, C = human-to-human transmission
