# Supplementary material for: Age-dependent virulence of human pathogens
Source: PLoS Pathog. 2022 Sep 22;18(9):e1010866. doi: 10.1371/journal.ppat.1010866 (PMC9531802; doi:10.1371/journal.ppat.1010866)
Supplement: S5 Table — The table reports the estimates (with SE and 95% CI), z and P values for the parameters retained in the model with the lowest BIC values. Number of observations = 443; number of deaths/ number of cases = 54,782/3,884764. (DOCX) [file ppat.1010866.s005.docx]

S5 Table. Finite mixture model with a beta-binomial distribution of errors exploring the effect of length of human-pathogen association, animal reservoir, and human-to-human transmission on age-specific CFR (number of deaths/number of cases) for the restricted dataset including only viral diseases. The table reports the estimates (with SE and 95% CI), z and P values for the parameters retained in the model with the lowest BIC values. Number of observations = 443; number of deaths/ number of cases = 54,782/3,884764.

| *Effects* | *Estimate (SE)* | *95% CI* | *z* | *P* |
| --- | --- | --- | --- | --- |
| Intercept | 0.974 (0.435) | 0.121/1.826 | 2.24 | 0.0253 |
| Age | -0.016 (0.055) | -0.124/0.092 | -0.29 | 0.7725 |
| Age^2^ | 0.007 (0.003) | 0.001/0.013 | 2.37 | 0.0179 |
| Date | -0.496 (0.069) | -0.630/-0.361 | -7.21 | <0.0001 |
| Intertropical (no)  (yes) | -0.757 (0.158)  0 | -1.066/-0.449 | -4.81 | <0.0001 |
| Length of association (ancient)  (emerging) | -0.224 (0.255)  0 | -0.7240/0.277 | -0.88 | 0.3808 |
| Animal reservoir (no)  (yes) | -0.204 (0.140)  0 | -0.480/0.071 | -1.46 | 0.1454 |
| Human to human transmission (no)  (yes) | -0.071 (0.221)  0 | -0.505/0.362 | -0.32 | 0.7468 |
| Age x length of association (ancient)  (emerging) | -0.095 (0.025)  0 | -0.143/-0.046 | -3.83 | 0.0001 |
