# Supplementary material for: Age-dependent virulence of human pathogens
Source: PLoS Pathog. 2022 Sep 22;18(9):e1010866. doi: 10.1371/journal.ppat.1010866 (PMC9531802; doi:10.1371/journal.ppat.1010866)
Supplement: S6 Table — We report the -2 Log Likelihood, AIC, BIC, Pearson Statistics, number of parameters (k), the overdispersion parameter (Pearson Statistic/(N-k), and the ΔBIC. N = 873 observations. We ran 35 competitive finite mixture models. The model with the lowest BIC value is highlighted in green. (DOCX) [file ppat.1010866.s006.docx]

S6 Table. Model comparison on the effect of transmission by body fluids, ingestion, inhalation, vectors on age specific CFR for 28 human infectious diseases. We report the -2 Log Likelihood, AIC, BIC, Pearson Statistics, number of parameters (k), the overdispersion parameter (Pearson Statistic/(N-k), and the ΔBIC. N = 873 observations. We ran 35 competitive finite mixture models. The model with the lowest BIC value is highlighted in green.

|  | **-2 Log Likelihood** | **AIC** | **BIC** | **Pearson Statistic** | **k** | **Pearson Statistic/(N-k)** | **ΔBIC** |
| --- | --- | --- | --- | --- | --- | --- | --- |
| *Main factors* |  |  |  |  |  |  |  |
| 1. Intercept | 8505.2 | 8509.2 | 8518.7 | 883.4 | 2 | 1.014 | 359.6 |
| 1. Age | 8478.4 | 8484.4 | 8498.7 | 870.9 | 3 | 1.001 | 339.6 |
| 1. Age + Age² | 8473.9 | 8481.9 | 8500.9 | 875.1 | 4 | 1.007 | 341.8 |
| 1. Age + Age² + Date + Intertropical | 8232.7 | 8244.7 | 8273.3 | 847.6 | 6 | 0.978 | 114.2 |
| 1. Age + Age² + Date + Intertropical + A + B + C + D | 8091.4 | 8111.4 | 8159.1 | 801 | 10 | 0.928 | 0 |
|  |  |  |  |  |  |  |  |
| *Interactions between Age and A,B,C,D* |  |  |  |  |  |  |  |
| 1. 4 + Age * A + Age * B + Age * C + Age * D | 8085 | 8113 | 8179.8 | 800.4 | 14 | 0.932 | 20.7 |
| 1. 4 + Age * A + Age * B + Age * C | 8090 | 8116 | 8178.1 | 802.6 | 13 | 0.933 | 19 |
| 1. 4 + Age * A + Age * B + Age * D | 8085.3 | 8111.3 | 8173.3 | 801.3 | 13 | 0.931 | 14.2 |
| 1. 4 + Age * A + Age * C + Age * D | 8088.4 | 8114.4 | 8176.4 | 802.2 | 13 | 0.933 | 17.3 |
| 1. 4 + Age * B + Age * C + Age * D | 8084.6 | 8110.6 | 8172.6 | 799.5 | 13 | 0.929 | 13.5 |
| 1. 4 + Age * A + Age * B | 8090.4 | 8114.4 | 8171.6 | 800.9 | 12 | 0.93 | 12.5 |
| 1. 4 + Age * A + Age * C | 8090.4 | 8114.4 | 8171.7 | 805.7 | 12 | 0.936 | 12.6 |
| 1. 4 + Age * A + Age * D | 8088.7 | 8112.7 | 8170 | 800.9 | 12 | 0.93 | 10.9 |
| 1. 4 + Age * B + Age * C | 8090.1 | 8114.1 | 8171.4 | 801.9 | 12 | 0.931 | 12.3 |
| 1. 4 + Age * B + Age * D | 8085.3 | 8109.3 | 8166.6 | 801.6 | 12 | 0.931 | 7.5 |
| 1. 4 + Age * C + Age * D | 8088.4 | 8112.4 | 8169.6 | 802.2 | 12 | 0.932 | 10.5 |
| 1. 4 + Age * A | 8091.3 | 8113.3 | 8165.8 | 801.5 | 11 | 0.93 | 6.7 |
| 1. 4 + Age * B | 8090.4 | 8112.4 | 8164.9 | 800.5 | 11 | 0.929 | 5.8 |
| 1. 4 + Age * C | 8090.5 | 8112.5 | 8164.9 | 802.8 | 11 | 0.931 | 5.8 |
| 1. 4 + Age * D | 8088.7 | 8110.7 | 8163.2 | 801 | 11 | 0.929 | 4.1 |
|  |  |  |  |  |  |  |  |
| *Interactions between Age² and A,B,C,D* |  |  |  |  |  |  |  |
| 1. 5 + Age² * A + Age² * B + Age² * C + Age² * D | 8094.4 | 8130.4 | 8216.3 | 813 | 18 | 0.951 | 57.2 |
| 1. 5 + Age² * A + Age² * B + Age² * C | 8082.2 | 8116.2 | 8197.3 | 802.1 | 17 | 0.937 | 38.2 |
| 1. 5 + Age² * A + Age² * B + Age² * D | 8082.7 | 8116.7 | 8203.9 | 803 | 17 | 0.938 | 38.8 |
| 1. 5 + Age² * A + Age² * C + Age² * D | 8088.7 | 8122.7 | 8196.8 | 795.3 | 17 | 0.929 | 44.8 |
| 1. 5 + Age² * B + Age² * C + Age² * D | 8081.7 | 8115.7 | 8191.1 | 801.5 | 17 | 0.936 | 37.7 |
| 1. 5 + Age² * A + Age² * B | 8082.8 | 8114.8 | 8193 | 803 | 16 | 0.937 | 32 |
| 1. 5 + Age² * A + Age² * C | 8084.7 | 8116.7 | 8192.7 | 802.5 | 16 | 0.936 | 33.9 |
| 1. 5 + Age² * A + Age² * D | 8084.3 | 8116.3 | 8190.5 | 799.7 | 16 | 0.933 | 33.6 |
| 1. 5 + Age² * B + Age² * C | 8082.2 | 8114.2 | 8207.9 | 802.2 | 16 | 0.936 | 31.4 |
| 1. 5 + Age² * B + Age² * D | 8099.6 | 8131.6 | 8193 | 829.1 | 16 | 0.967 | 48.8 |
| 1. 5 + Age² * C + Age² * D | 8084.6 | 8116.6 | 8186.1 | 798.3 | 16 | 0.932 | 33.9 |
| 1. 5 + Age² * A | 8084.5 | 8114.5 | 8188 | 799.1 | 15 | 0.931 | 27 |
| 1. 5 + Age² * B | 8086.4 | 8116.4 | 8186.1 | 809 | 15 | 0.943 | 28.9 |
| 1. 5 + Age² * C | 8084.5 | 8114.5 | 8185.9 | 798.9 | 15 | 0.931 | 27 |
| 1. 5 + Age² * D | 8084.3 | 8114.3 | 8179.8 | 799.4 | 15 | 0.932 | 26.8 |

A = body fluids, B = ingestion, C = inhalation, D = vector
