# Supplementary material for: Age-dependent virulence of human pathogens
Source: PLoS Pathog. 2022 Sep 22;18(9):e1010866. doi: 10.1371/journal.ppat.1010866 (PMC9531802; doi:10.1371/journal.ppat.1010866)
Supplement: S7 Table — We report the results for the linear (regression model analysis) and the non-linear (smoothing model analysis) components. The model was run using a binomial distribution of errors (number of deaths/number of cases). (DOCX) [file ppat.1010866.s007.docx]

S7 Table. Generalized additive model (GAM) investigating the shape of the relationship between COVID-19 CFR and age in women and men. We report the results for the linear (regression model analysis) and the non-linear (smoothing model analysis) components. The model was run using a binomial distribution of errors (number of deaths/number of cases).

|  | **Regression model analysis** | | | **Smoothing model analysis** | |
| --- | --- | --- | --- | --- | --- |
| *Parameter* | *Estimate (SE)* | *t* | *p* | *χ2* | *p* |
| Sex (M) | 0.492 (0.176) | 2.79 | 0.0097 |  |  |
| Age | 0.621 (0.009) | 67.57 | <0.0001 | 138.92 | <0.0001 |
| Age x Sex (M) | 0.014 (0.011) | 1.27 | 0.2161 | 9.92 | 0.0192 |
