# Supplementary material for: Age-dependent virulence of human pathogens
Source: PLoS Pathog. 2022 Sep 22;18(9):e1010866. doi: 10.1371/journal.ppat.1010866 (PMC9531802; doi:10.1371/journal.ppat.1010866)
Supplement: S8 Table — We report the -2 Log Likelihood, AIC, BIC, Pearson Statistics, number of parameters (k), the overdispersion parameter (Pearson Statistic/(N-k), and the ΔBIC. N = 873 observations. We ran 19 competitive finite mixture models to identify the model with the lowest BIC. Only models with the main effects (no interaction) were compared here. The model with the lowest BIC value is highlighted in green. (DOCX) [file ppat.1010866.s008.docx]

S8 Table. Model comparison on the effect of transmission by body fluids, ingestion, inhalation, vectors on age specific CFR for 28 human infectious diseases. We report the -2 Log Likelihood, AIC, BIC, Pearson Statistics, number of parameters (k), the overdispersion parameter (Pearson Statistic/(N-k), and the ΔBIC. N = 873 observations. We ran 19 competitive finite mixture models to identify the model with the lowest BIC. Only models with the main effects (no interaction) were compared here. The model with the lowest BIC value is highlighted in green.

|  | **-2 Log Likelihood** | **AIC** | **BIC** | **Pearson Statistic** | **k** | **Pearson Statistic/(N-k)** | **ΔBIC** |
| --- | --- | --- | --- | --- | --- | --- | --- |
| *Main factors* |  |  |  |  |  |  |  |
| 1. Intercept | 8505.2 | 8509.2 | 8518.7 | 883.4 | 2 | 1.014 | 365.1 |
| 1. Age | 8478.4 | 8484.4 | 8498.7 | 870.9 | 3 | 1.001 | 345.1 |
| 1. Age + Age² | 8473.9 | 8481.9 | 8500.9 | 875.1 | 4 | 1.007 | 347.3 |
| 1. Age + Age² + Date + Intertropical | 8232.7 | 8244.7 | 8273.3 | 847.6 | 6 | 0.978 | 119.7 |
| 1. Age + Age² + Date + Intertropical + A + B + C + D | 8091.4 | 8111.4 | 8159.1 | 801 | 10 | 0.928 | 5.5 |
| 1. Age + Age² + Date + Intertropical + A + B + C | 8121.7 | 8139.7 | 8182.6 | 810.7 | 9 | 0.938 | 29 |
| 1. Age + Age² + Date + Intertropical + A + B + D | 8098.5 | 8116.5 | 8159.5 | 803.9 | 9 | 0.930 | 5.9 |
| 1. Age + Age² + Date + Intertropical + A + C + D | 8097.3 | 8115.3 | 8158.3 | 783.4 | 9 | 0.907 | 4.7 |
| 1. Age + Age² + Date + Intertropical + B + C + D | 8222.9 | 8240.9 | 8283.8 | 881.8 | 9 | 1.021 | 130.2 |
| 1. Age + Age² + Date + Intertropical + A + B | 8121.9 | 8137.9 | 8176.1 | 809.2 | 8 | 0.935 | 22.5 |
| 1. Age + Age² + Date + Intertropical + A + C | 8123.5 | 8139.5 | 8177.6 | 822.3 | 8 | 0.951 | 24 |
| 1. Age + Age² + Date + Intertropical + A + D | 8099.4 | 8115.4 | 8153.6 | 794.3 | 8 | 0.918 | 0 |
| 1. Age + Age² + Date + Intertropical + B + C | 8232.6 | 8248.6 | 8286.8 | 846.5 | 8 | 0.979 | 133.2 |
| 1. Age + Age² + Date + Intertropical + B + D | 8227.5 | 8243.5 | 8281.7 | 850.6 | 8 | 0.983 | 128.1 |
| 1. Age + Age² + Date + Intertropical + C + D | 8227.7 | 8243.7 | 8281.8 | 841.5 | 8 | 0.973 | 128.2 |
| 1. Age + Age² + Date + Intertropical + A | 8123.5 | 8137.5 | 8170.9 | 823.5 | 7 | 0.951 | 17.3 |
| 1. Age + Age² + Date + Intertropical + B | 8232.6 | 8246.6 | 8280 | 846.1 | 7 | 0.977 | 126.4 |
| 1. Age + Age² + Date + Intertropical + C | 8232.6 | 8246.6 | 8280 | 847 | 7 | 0.978 | 126.4 |
| 1. Age + Age² + Date + Intertropical + D | 8228.2 | 8242.2 | 8275.6 | 842.9 | 7 | 0.973 | 122 |

A = body fluids, B = ingestion, C = inhalation, D = vector
