# Supplementary material for: Age-dependent virulence of human pathogens
Source: PLoS Pathog. 2022 Sep 22;18(9):e1010866. doi: 10.1371/journal.ppat.1010866 (PMC9531802; doi:10.1371/journal.ppat.1010866)
Supplement: S9 Table — The table reports the estimates (with SE and 95% CI), z and P values for the parameters retained in the model with the lowest BIC value. Number of observations = 873; number of deaths/ number of cases = 143877/5624790. (DOCX) [file ppat.1010866.s009.docx]

S9 Table. Finite mixture model with a beta-binomial distribution of errors exploring the effect of transmission mode (contact with body fluids, ingestion, inhalation, vector) on age-specific number of deaths/number of cases. The table reports the estimates (with SE and 95% CI), z and P values for the parameters retained in the model with the lowest BIC value. Number of observations = 873; number of deaths/ number of cases = 143877/5624790.

| *Effects* | *Estimate (SE)* | *95% CI* | *z* | *P* |
| --- | --- | --- | --- | --- |
| Intercept | 3.279 (0.259) | 2.770/3.787 | 12.64 | <0.0001 |
| Age | -0.041 (0.034) | -0.108/0.025 | -1.22 | 0.2210 |
| Age^2^ | 0.007 (0.002) | 0.003/0.011 | 3.27 | 0.0011 |
| Date | -0.615 (0.037) | -0.686/-0.543 | -16.87 | <0.0001 |
| Intertropical (no)  (yes) | -0.498 (0.103)  0 | -0.699/-0.296 | -4.83 | <0.0001 |
| Body fluids (no)  (yes) | -2.093 (0.172)  0 | -2.430/-1.756 | -12.18 | <0.0001 |
| Vector (no)  (yes) | -0.465 (0.093)  0 | -0.646/-0.283 | -5.02 | <0.0001 |
