# Supplementary material for: Age-dependent virulence of human pathogens
Source: PLoS Pathog. 2022 Sep 22;18(9):e1010866. doi: 10.1371/journal.ppat.1010866 (PMC9531802; doi:10.1371/journal.ppat.1010866)

S1 Fig. Heatmap of the association (contingency coefficients) between explanatory variables considered here. The redder the color the stronger the association. A Fisher’s exact test with a sequential Bonferroni correction showed that only the associations between pathogen type and length of human-pathogen association (p = 0.0084) and between animal reservoir and vector (p = 0.0058) were statistically significant.


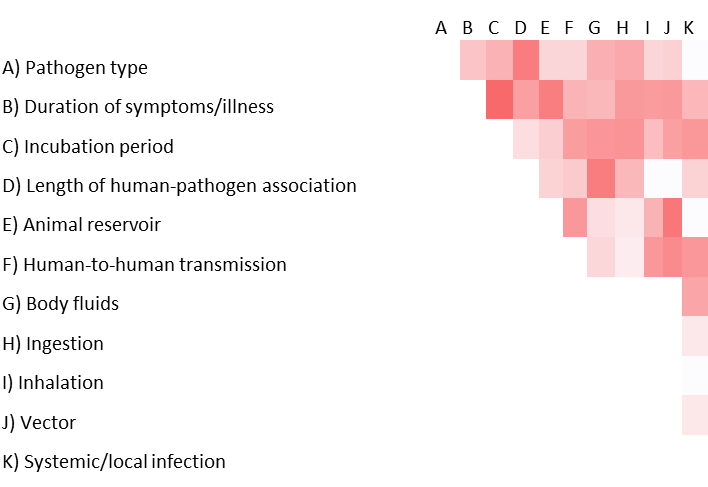

Supplement: S1 Fig — The redder the color the stronger the association. A Fisher’s exact test with a sequential Bonferroni correction showed that only the associations between pathogen type and length of human-pathogen association (p = 0.0084) and between animal reservoir and vector (p = 0.0058) were statistically significant. (DOCX) [file ppat.1010866.s010.docx]
