# Supplementary material for: Age-dependent virulence of human pathogens
Source: PLoS Pathog. 2022 Sep 22;18(9):e1010866. doi: 10.1371/journal.ppat.1010866 (PMC9531802; doi:10.1371/journal.ppat.1010866)

S2 Fig. Smoothing component analysis of the effect of age on CFR (number of deaths / number of cases) for the 28 human infectious diseases considered here. Each panel shows the spline fit (with the 95% CI) of generalized additive models run for each disease, after the linear trend has been removed. For instance, the fact that the spline fit for the relationship between CFR and age for AIDS is a flat line with 95% CI overlapping zero over the whole range of ages indicates that CFR only varies linearly with age. On the contrary, the spline fit for cholera shows a strong non-linear pattern.


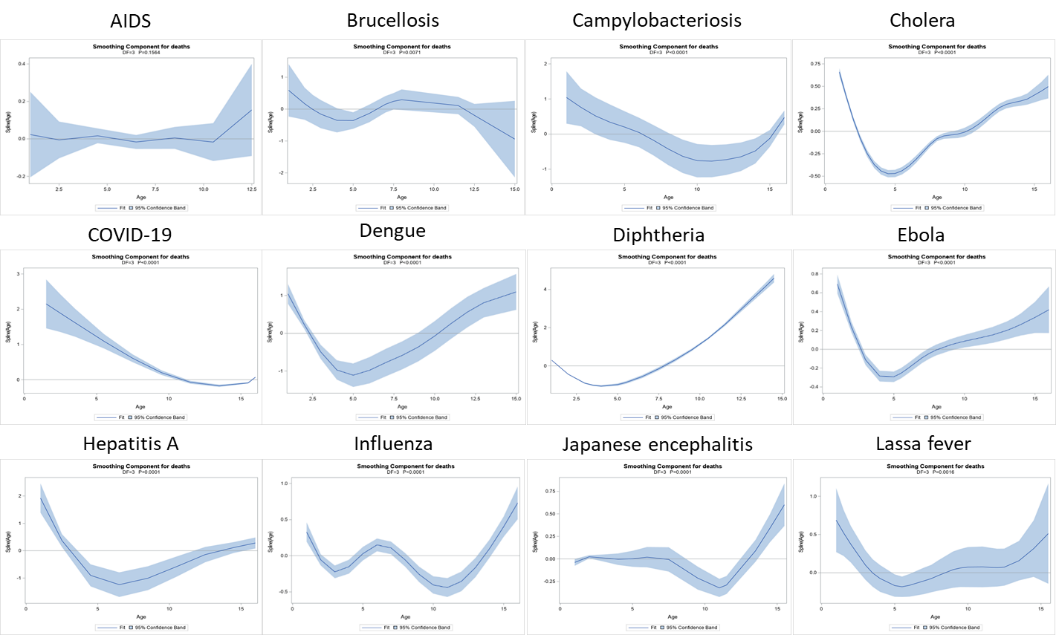

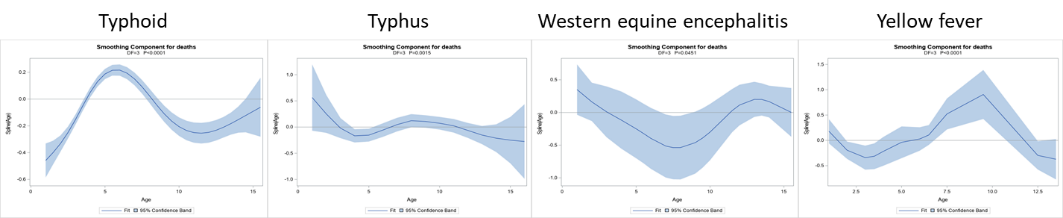


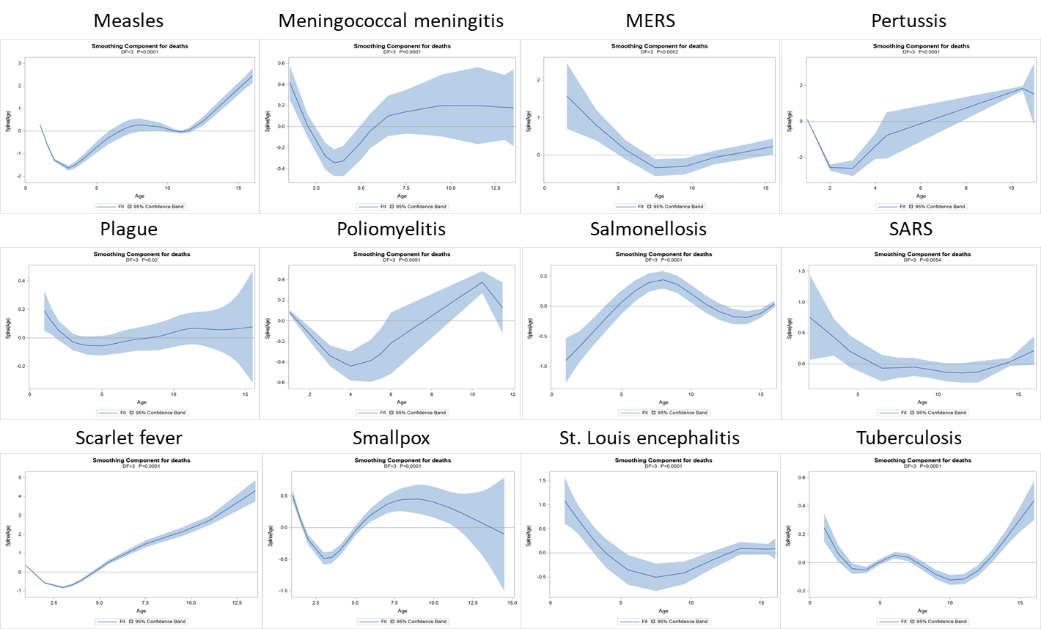

Supplement: S2 Fig — Each panel shows the spline fit (with the 95% CI) of generalized additive models run for each disease, after the linear trend has been removed. For instance, the fact that the spline fit for the relationship between CFR and age for AIDS is a flat line with 95% CI overlapping zero over the whole range of ages indicates that CFR only varies linearly with age. On the contrary, the spline fit for cholera shows a strong non-linear pattern. (DOCX) [file ppat.1010866.s011.docx]
