# Supplementary material for: Age-dependent virulence of human pathogens
Source: PLoS Pathog. 2022 Sep 22;18(9):e1010866. doi: 10.1371/journal.ppat.1010866 (PMC9531802; doi:10.1371/journal.ppat.1010866)

S3 Fig. Age-dependent variation of CFR (%) for COVID-19 in women (red dots and line) and men (blue dots and line). Each dot represents the percent of infected people dying (number of deaths/number of cases) in each age class. Bars represent the binomial 95% confidence intervals. The dotted lines represent the fit of the GAM. Multiple CFR values for a given age class refer to independent datasets.


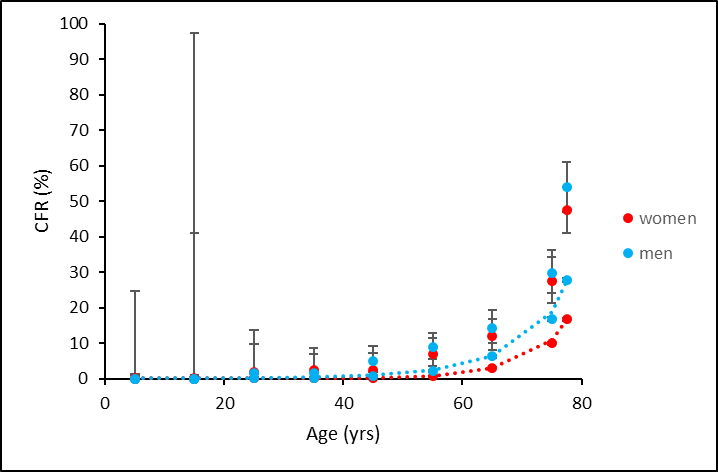

Supplement: S3 Fig — Each dot represents the percent of infected people dying (number of deaths/number of cases) in each age class. Bars represent the binomial 95% confidence intervals. The dotted lines represent the fit of the GAM. Multiple CFR values for a given age class refer to independent datasets. (DOCX) [file ppat.1010866.s012.docx]
