# Supplementary material for: Age-dependent virulence of human pathogens
Source: PLoS Pathog. 2022 Sep 22;18(9):e1010866. doi: 10.1371/journal.ppat.1010866 (PMC9531802; doi:10.1371/journal.ppat.1010866)

S4 Fig. CFR (%) for diseases transmitted through contact with body fluids (A), and diseases transmitted through vector bites (B). We report the mean ± SE.


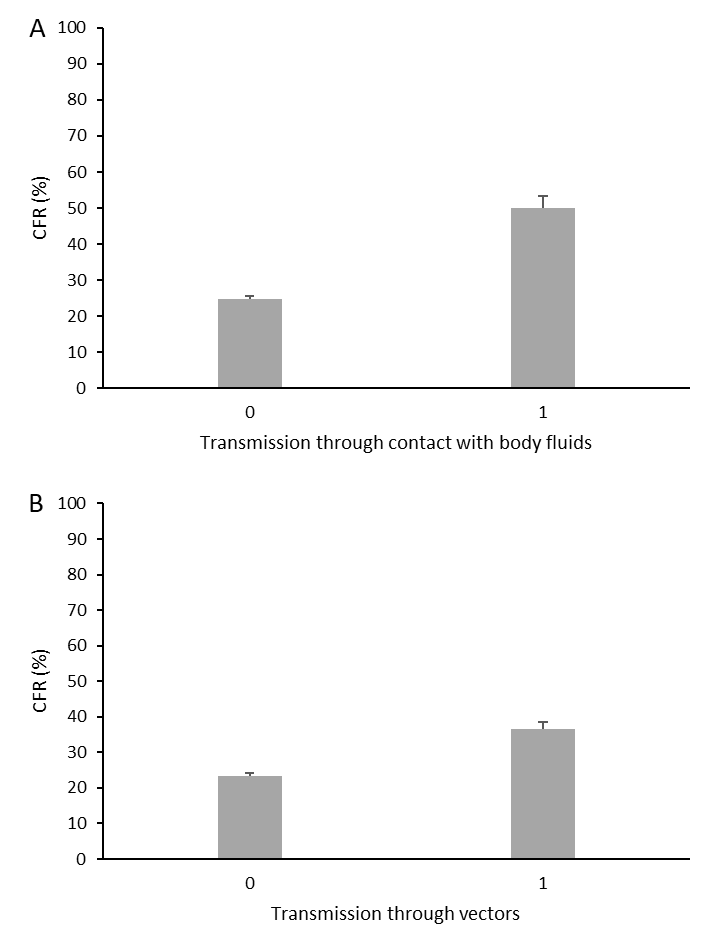

Supplement: S4 Fig — CFR (%) for diseases transmitted through contact with body fluids (A), and diseases transmitted through vector bites (B). We report the mean ± SE. (DOCX) [file ppat.1010866.s013.docx]
