# Supplementary material for: Age-dependent virulence of human pathogens
Source: PLoS Pathog. 2022 Sep 22;18(9):e1010866. doi: 10.1371/journal.ppat.1010866 (PMC9531802; doi:10.1371/journal.ppat.1010866)
Supplement: S1 Text — (DOCX) [file ppat.1010866.s014.docx]

SAS codes

/* Generalized additive model*/

ods graphics on;

**proc** **gam** plots=components(additive clm);

model deaths/cases = spline(age) /dist=binomial;

by disease;

output out=gam predicted;

**run**;

ods graphics off;

/* Beta-binomial model1 (full model) systemic=systemic infection (yes/no), illness=duration of symptoms, incubation = incubation period*/

**proc** **fmm** noitprint;

class intertropical systemic ;

model deaths/cases=age age2 date intertropical illness incubation systemic

age*illness age*incubation age*systemic

age2*illness age2*incubation age2*systemic/link=logit dist=betabinomial cl;

output out=fmm pred=pcfr;

**run**;

**quit**;

/* Beta-binomial model2 (full model) pathogen=pathogen type, association=length of human-pathogen association, reservoir=animal reservoir, hth=human-to-human transmission*/

**proc** **fmm** noitprint;

class intertropical pathogen association reservoir hth ;

model deaths/cases=age age2 date intertropical pathogen association reservoir hth

age*pathogen age*association age*reservoir age*hth

age2*pathogen age2*association age2*reservoir age2*hth/link=logit dist=betabinomial cl;

output out=fmm pred=pcfr;

**run**;

**quit**;

/* Beta-binomial model3 (full model) bodyfluids=transmission through contact with body fluids, ingestion=transmission by ingestion, inhalation=transmission by inhalation, vector=transmission through vectors */

**proc** **fmm** noitprint;

class intertropical bodyfluids ingestion inhalation vector ;

model deaths/cases=age age2 date intertropical bodyfluids ingestion inhalation vector

age*bodyfluids age*ingestion age*inhalation age*vector

age2*bodyfluids age2*ingestion age2*inhalation age2*vector/link=logit dist=betabinomial cl;

output out=fmm pred=pcfr;

**run**;

**quit**;
